# Supplementary material for: The interactome of intact mitochondria by cross-linking mass spectrometry provides evidence for coexisting respiratory supercomplexes
Source: Mol Cell Proteomics. 2017 Dec 8;17(2):216–32. doi: 10.1074/mcp.RA117.000470 (PMC5795388; doi:10.1074/mcp.RA117.000470)
Supplement: Supplemental Data [file supp_RA117.000470_133922_0_supp_23978_fzffwf.zip › spectra_annotation/mito_spectra_annotation/500-1-13-1-24-1.pdf]

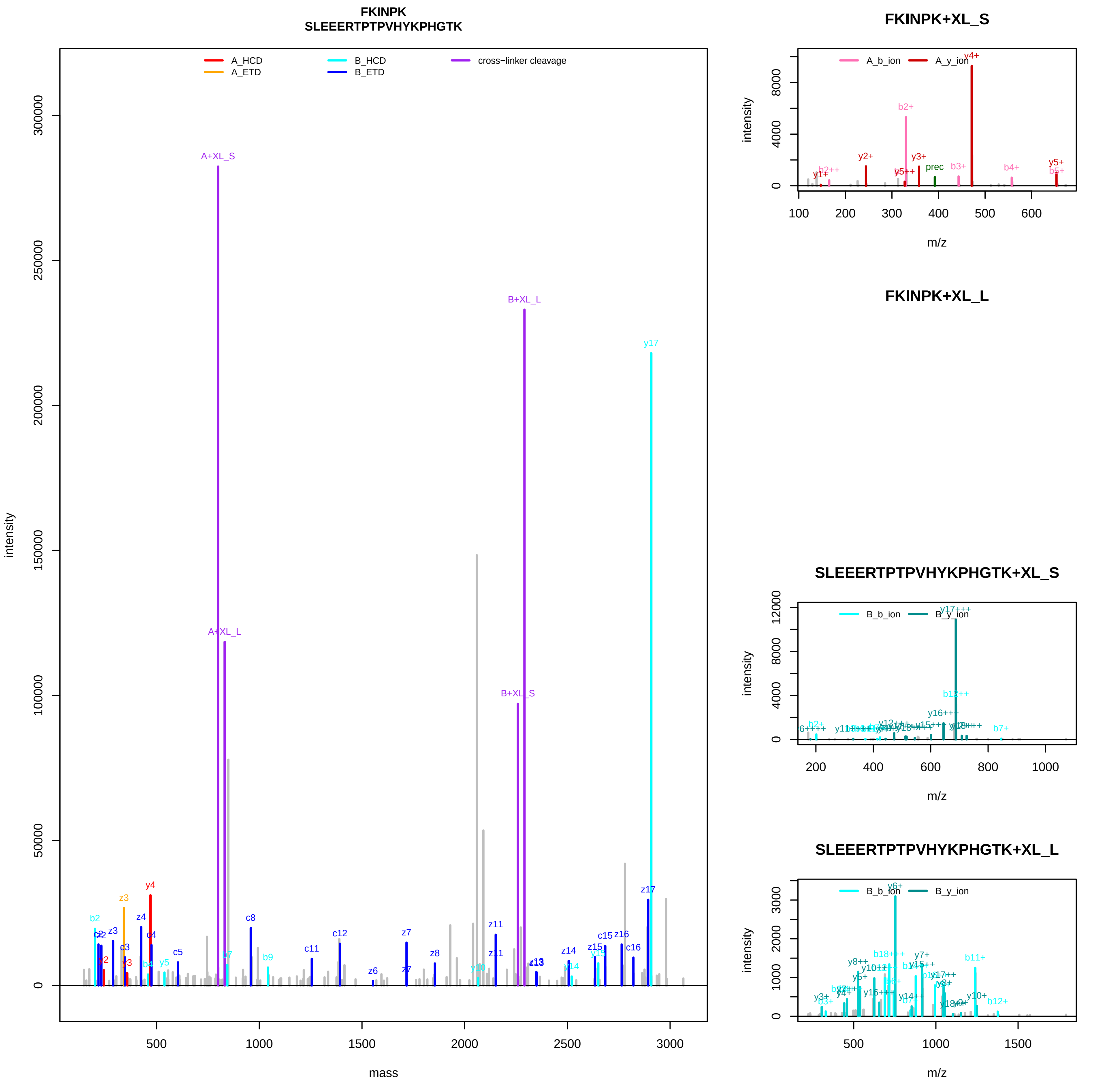
FKINPK
SLEEERTPTPVHYKPHGTK
A_HCD
A_ETD
B_HCD
B_ETD
cross−linker cleavage
A+XL_S
A+XL_L
B+XL_L
B+XL_S
y17
z17
y4
z3
b2
c2
z2
z3
y2
y3
c3
z4
c4
b4
y5
c5
b7
c8
b9
c11
c12
z6
z7
z8
z11
z13
z14
z15
c15
z16
c16
intensity
mass
0
50000
100000
150000
200000
250000
300000
500
1000
1500
2000
2500
3000
FKINPK+XL_S
A_b_ion
A_y_ion
y4+
b2+
y2+
y3+
prec
b3+
b4+
y5+
y1+
intensity
m/z
FKINPK+XL_L
SLEEERTPTPVHYKPHGTK+XL_S
B_b_ion
B_y_ion
y17+++
b12++
y16+++
b7+
b2+
intensity
m/z
SLEEERTPTPVHYKPHGTK+XL_L
B_b_ion
B_y_ion
y6+
y8++
y7+
b11+
b12+
y10+
y3+
intensity
m/z
